# A cash transfer plus gender transformative economic empowerment intervention seeking to improve the wellbeing of caregivers of children and adolescents living with HIV in South Africa: a feasibility study protocol for a pilot cluster randomized trial

**DOI:** 10.1186/s40814-025-01643-3

**Published:** 2025-04-23

**Authors:** Darshini Govindasamy, Nwabisa Shai, Nelly Mwandacha, Stanley Carries, Nokwanda Sithole, Kalysha Closson, Arvin Bhana, Lovemore Sigwadhi, Laura Washington, Andrew Gibbs, Angela Kaida

**Affiliations:** 1 South African Medical Research Council, Durban, South Africa; 2 School of Public Health, Faculty of Health Sciences, University of the Witwatersrand, Johannesburg, South Africa; 3 Simon Fraser University, Burnaby, Canada; 4 University of British Columbia, Vancouver, Canada; 5 Stellenbosch University, Stellenbosch, South Africa; 6 Project Empower, Durban, South Africa; 7 University of Exeter, Exeter, UK; 8 University College London, London, UK; 9 University of KwaZulu-Natal, Durban, South Africa

**Keywords:** Caregivers, Wellbeing, HIV/AIDS, Economic empowerment, Gender transformative intervention, South Africa

## Abstract

**Background:**

In sub-Saharan Africa, HIV care is dependent on informal caregiving, typically by female family members. Informal caregiving has been associated with numerous negative effects on caregivers (i.e., depression, intimate partner violence (IPV), financial insecurity). These factors impact caregivers’ ability to provide care and their own wellbeing. South Africa is home to approximately 17% of the world’s children and adolescents living with HIV (CALHIV), making the development of initiatives that mitigate the negative effects of caregiving critical. This protocol details the design of a cluster randomized trial seeking to assess the feasibility, preliminary effectiveness, cost-effectiveness, and acceptability of a cash transfer plus gender transformative economic empowerment intervention for improving psychological wellbeing, depressive symptoms, gender equality, and economic outcomes of caregivers of CALHIV.

**Methods:**

Caregivers of CALHIV will be recruited from public sector HIV clinics within the eThekwini Municipality, KwaZulu-Natal, South Africa. Clusters will be randomly assigned to intervention or control groups. Participants in the intervention arm (*n* = 120) will receive cash transfers (ZAR350, USD $18.79) and enroll in a program (10 workshop sessions) over a 6-month period. Participants in the control arm (*n* = 120) will receive a monthly cash transfer (ZAR350, USD $18.79) for a 6-month period and a once-off standard mobile message, encouraging linkage to healthcare services. Participants will be interviewed at baseline and endline, at the 7-month mark, to collect socio-demographic, health and wellbeing status, IPV, costs and earnings, and food security data. The primary outcomes include consent rate, overall retention rate, workshops retention rate, cash transfer protocol adherence, staff perceptions on implementation, psychological wellbeing, depressive symptoms, and IPV. A qualitative study and economic evaluation will be conducted alongside the main trial to probe participant perceptions of the intervention and assess cost and cost-effectiveness.

**Discussion:**

This trial has the potential to inform a larger confirmatory trial which will be valuable for informing post-pandemic recovery efforts for caregivers of CALHIV and others disproportionally burdened by compounding health and social crises.

**Trial registration:**

PACTR202311618532061. Registry name: Pan African Clinical Trial Registry (PACTR), retrospectively registered on November 21, 2023; The first participant was enrolled on August 24, 2023.

**Supplementary Information:**

The online version contains supplementary material available at 10.1186/s40814-025-01643-3.

## Background

South Africa has the largest HIV epidemic globally, with approximately 17% of the estimated 2.58 million children and adolescents living with HIV (CALHIV) living there and thus a similar number of caregivers [1]. Supportive caregivers are critical to the health and wellbeing of CALHIV [2]. HIV care for CALHIV in resource-poor settings is highly dependent on informal caregiving, usually delivered by a mother or female family member [3]. However, informal caregiving has been linked to numerous negative effects on the caregivers themselves. Unpaid caregiving is associated with depression and intimate partner violence (IPV) and can result in long-term negative economic consequences [4, 5]. Moreover, as informal caregiving is highly gendered and often occurs in lower income households, these negative impacts are amplified by social inequities such as gender and income inequality [6]. These factors impact both caregivers’ own wellbeing and their ability to provide quality care to CALHIV [2].

The COVID- 19 pandemic magnified gender and income inequalities and is predicted to have a long-term health and socio-economic impacts [7, 8]. To mitigate the economic impacts of the COVID- 19 pandemic restrictions on the most vulnerable, the South African government implemented the COVID- 19 Social Relief of Distress (SRD) grant [9]. Introduced in May 2020 and set to continue as a basic income grant in the future, this social grant comprises a monthly ZAR 350 payment (USD $18.79) delivered over a 6-month period [9]. Modeling studies have noted the benefits grants such as the SRD grant could have on the wellbeing of the most marginalized, including unpaid workers, during pandemic recovery and if made permanent [10–12]. However, while the United Nations Development Programme’s strategy for pandemic recovery has incorporated enhancing social protection into the next phases of its COVID- 19 crisis response, there is a notable lack of empirical research on pandemic recovery strategies and social protection in South Africa [12]. There is a need to enhance national efforts surrounding pandemic recovery strategies and ensure approaches empower the most vulnerable.

The wellbeing of women caregivers of CALHIV in South Africa can be improved through strategies that strengthen their economic livelihoods by enabling women to generate income, improve self-esteem, achieve valued social status, and increase autonomy and control over their lives through access to resources [13–15]. In 2021, our team piloted a randomized controlled trial of an economic incentive package intended to improve the wellbeing of caregivers of adolescents living with HIV in KwaZulu-Natal, South Africa, during COVID- 19 by addressing key barriers (CWeL trial) [16]. The economic incentive package comprised a monthly ZAR350 (USD $18.79) cash transfer paired with motivational text messages that promoted caregiver wellbeing and were delivered over a 3-month period [16]. The CWeL trial was associated with increases in psychological wellbeing and decreases in depressive symptoms and had a significant effect on reducing caregiver-related distress [16]. Furthermore, caregivers expressed the need for group-based psychosocial and economic empowerment support to enhance the intervention [16]. Group-based economic empowerment training interventions are another approach to strengthening livelihoods [17]. When economic empowerment interventions are combined with components seeking to transform gender roles to ones that are more equitable, termed gender transformative approaches, group-based economic empowerment training interventions can reduce women’s experiences of IPV and gender inequalities by addressing the power dynamics, social norms, attitudes, and social systems that underlie them [17–20]. Stepping stones and creating futures (SSCF) is a group-based gender transformative + economic livelihoods intervention that aims to address multiple drivers of IPV amongst youth in KwaZulu-Natal, South Africa, by transforming gender attitudes and strengthening livelihoods [17]. In a large randomized controlled trial led by the team, SSCF was associated with significant improvements in women’s livelihoods, and for those who attended more sessions, reductions in depressive symptoms [17]. Following on from and building off the CWeL trial and SSCF trial, the Caregiver Wellbeing Plus (CWeL +) project aims to co-develop a cash transfer plus (+) gender transformative economic empowerment intervention for improving the psychological wellbeing (positive mental health), gender equality, and economic outcomes of caregivers of CALHIV in KwaZulu-Natal, South Africa. As per the SSCF intervention, the economic empowerment program of the CWEL + intervention will be delivered to groups of caregivers from sampled clinics. Hence, a cluster-randomized trial (CRT) would be an appropriate evaluation design to assess the effects of the CWEL + intervention. However, full-scale CRTs are expensive for resource constrained settings and careful planning is needed. Thus, we will conduct a pilot CRT to understand the feasibility of implementing a larger trial of this nature, the applicability of the intervention to caregivers, and to obtain preliminary clinical outcome data to inform the design and sample size of a future full-scale trial [21].

### Objectives

- To co-develop a gender transformative economic empowerment intervention and a cash transfer to women caregivers of CALHIV.
- To assess the feasibility of this intervention in terms of key trial processes in the intervention and control arms.
- To describe participants’ views on the acceptability of delivery, content, safety, and utilization of the intervention versus control activities.
- To understand the mechanisms through which the intervention works to improve psychological wellbeing, economic empowerment, and experiences of gender inequity.
- To estimate the potential effect size of this intervention on key indicators to inform future trial design (improvements to psychological wellbeing, positive gender attitudes and earnings, and decreases in depressive symptoms and IPV) amongst caregivers of CALHIV.
- To measure the cost and cost-effectiveness of this intervention.

## Methodology

**Fig. 1:**
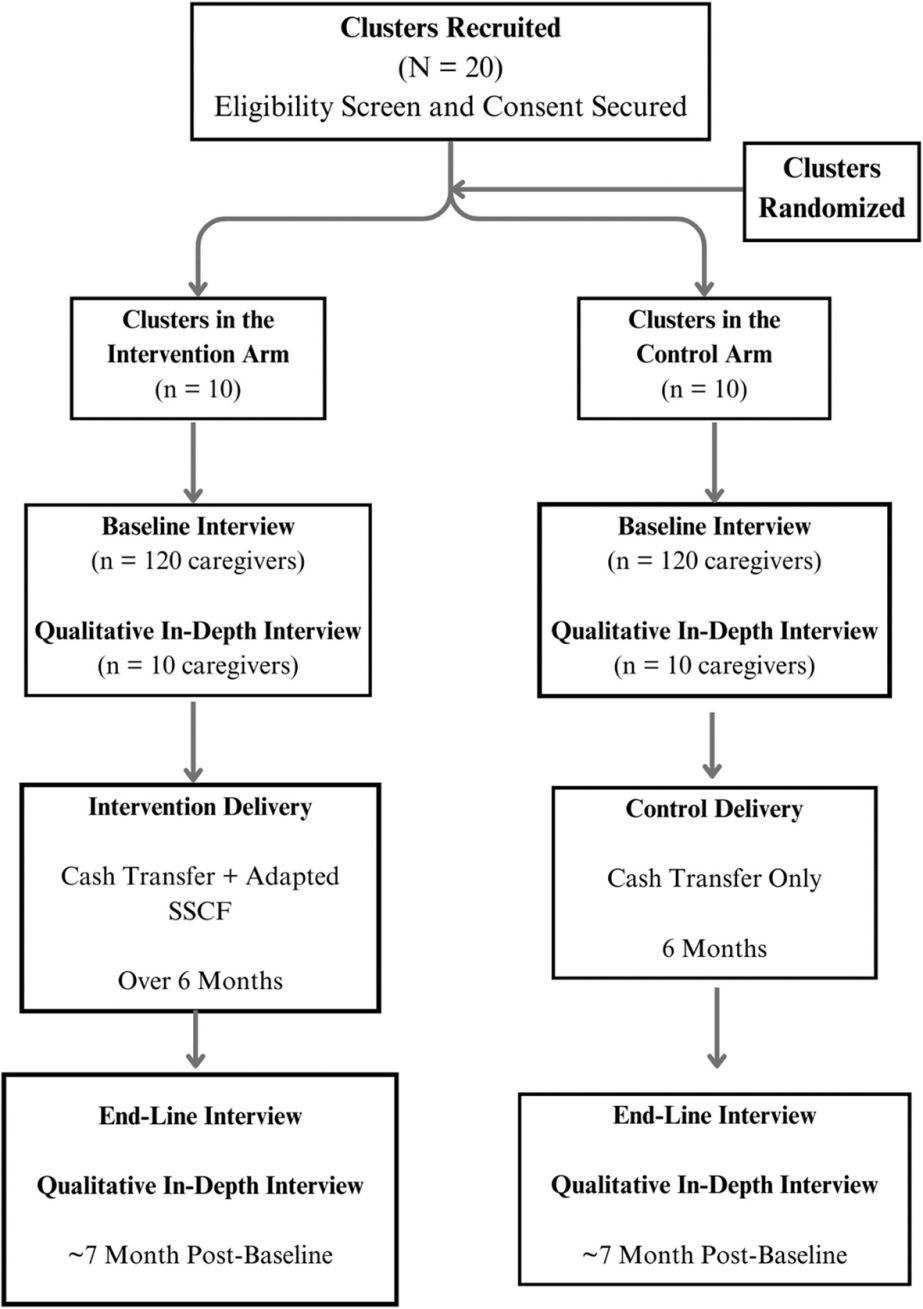
Standard protocol items: recommendation for intervention trials (SPIRIT) flow diagram

Reporting and methodology for the proposed study follow the Standard Protocol Items Recommendations for Interventional Trials (SPIRIT) [22] (see Fig. 1) and the Consolidated Standards of Reporting Trials (CONSORT)—extension to randomized pilot and feasibility trials [23] (see Additional file 1).

### Design

This will be a two-arm CRT comprising a quantitative survey, an economic evaluation, and a qualitative sub-study. Clusters (public-sector HIV clinics) will be randomized into an intervention arm (*N* = 10 cluster) and a control arm (*N* = 10 clusters).

### Setting and cluster eligibility

This study will be conducted in the largely peri-urban eThekwini Metropolitan Health District, in KwaZulu-Natal, South Africa. The eThekwini municipality includes a sampling frame of 35 government (public sector) HIV clinics within primary healthcare clinics, community healthcare centers, and outpatient hospital-based facilities. Approximately *N* = 20 HIV clinics (clusters) will be sampled. Clusters within a 25-km radius of the field office, providing antiretroviral therapy to *n* > 1000 patients, and previously involved in the CWeL or SSCF trials will be eligible to participate in this trial. Clusters who are already enrolled in an existing CRT mental health or gender-based violence (GBV) intervention will be ineligible for participation. Two clinics from nearby wards (geopolitical sub-divisions) will be selected and randomly assigned to the intervention or control arm.

### Participant eligibility criteria

Participants will be recruited from the *N* = 20 clusters (approximately 12 participants per clinic resulting in 120 participants per arm). Eligible participants for this trial will be adult (18 +) women primary caregivers of CALHIV (aged 5–19 years) who are eligible for South Africa’s COVID- 19 SRD grant but are not currently receiving it, have access to a mobile phone, and provide informed consent. See Additional file 2 for SRD grant eligibility criteria.

### Participant recruitment

Caregivers of CALHIV who are not receiving a COVID- 19 SRD grant will be identified by clinic staff on the day of their routine HIV care appointment. Clinic staff will then refer potential participants to trial fieldworkers, who will conduct a face-to-face preliminary screen, during or after their clinic appointment to ensure eligibility criteria are met. Potentially eligible participants will be invited to the trial’s field office whereby fieldworkers will explain the study in participants’ preferred language and verify their understanding of the study using a comprehension checklist, detailing challenges, randomization, and consent procedures. Should caregivers have a good understanding of the study and provide written consent, they will be enrolled in the trial.

### Baseline and endline assessment

After the screening, female field staff will administer a baseline questionnaire to participants in their preferred language (English or isiZulu) (see Additional file 3). Seven months post-intervention, an endline assessment will be conducted. The questionnaires will ask about participant demographics, health-related quality of life, psychological wellbeing, depressive symptoms, IPV, gender attitudes, gender equality, economic empowerment, costs and earnings, food security, stigma and discrimination, and social support. The endline questionnaire will repeat all measures and also feature a module to probe participant’s perceptions and experiences of the trial. Data from these questionnaires will be captured on REDCap (v11.0.3) using electronic tablets [24, 25].

### Cluster randomization

Approximately 20 clusters will be recruited, with 12 participants per cluster and 10 clusters per arm resulting in 120 participants per arm. For each selected ward/s, the statistician will use a random number generator to assign one cluster to the intervention arm and one cluster to the control arm.

### Blinding

The statisticians and senior investigators will be blinded to the arm allocation. Field staff involved in intervention or control delivery and assessments will not be blinded. Participants will not be blinded to study arm allocation.

### Intervention co-development

Before commencing the pilot CRT phase, a co-development phase was conducted. Three participatory workshops were conducted with a group of women caregivers of CALHIV from an existing caregiver advisory board (CAB). Led by Project Empower, a local feminist community-based NGO, these workshops facilitated the adaptation of the SSCF curriculum and training manuals to center the needs and preferences of the caregivers in the curriculum, training, implementation, and evaluation of the manuals and questionnaires. The gender analysis matrix [26], a participatory approach to intervention development that supports women in challenging gender assumptions constructively, was applied to ensure women’s needs were met and incorporated into the adaptation of SSCF. Investigators met regularly over a 2-month period to revise session manuals. Thereafter, a brief pre-test of the adapted workshop and data collection instruments, including the baseline and endline questionnaires, was conducted with a sample of caregivers. From this pre-test, the adapted workshop and data instruments were future refined and finalized.

### Intervention

Participants randomized to the intervention arm will receive a monthly ZAR350 (USD $18.79) cash transfer in addition to 10, 3-h face-to-face adapted SSCF workshop sessions over a 6-month period to assess the additional benefit of a short SSCF intervention irrespective of a monthly cash transfer. The workshops will be facilitated at the trial office by experienced facilitators from Project Empower. Topics featured in the workshops include maintaining positive mental health and wellbeing, parenting, caregiving for CALHIV, managing HIV-related stigma and HIV disclosure, and potential ways to effectively utilize the cash transfer to meet monthly financial goals that are locally appropriate. In addition, economic empowerment themes, such as seeking employment, budgeting, savings, and economic coercion, will be included to ameliorate participants’ economic livelihoods by incorporating activities that will help improve participants’ financial circumstances, decision-making power, and self-esteem. Cash will be transferred unconditionally once a month over the 6-month period via the SAMRC (South African Medical Research Council) ABSA CashSend system, a local electronic banking service used in the CWeL trial. Participants will receive one text message from the study team containing a PIN and one text message from ABSA bank containing a 10-digit withdrawal code; both the PIN and the code will be needed to withdraw the incentive payment from an ABSA ATM.

### Proposed theory of change

This intervention is hypothesized to increase psychological wellbeing and reduce depressive symptoms and IPV through four potential mechanisms: (1) increased sense of belonging and improved management of stress through the ability to fulfill caregiving responsibilities and improvement positive mental health coping strategies; (2) increased self-worth and agency through increased financial efficacy, engagement in income generation or saving activities, and increased decision-making power in relationships; (3) improved awareness of gender inequities and harmful gender norms; and (4) improved communication skills within relationships. See Additional file 4 for detailed theories of change from the CWeL and SSCF trials that have informed this trial [27].

### Control

Participants in the control arm will receive a monthly ZAR350 (USD $18.79) cash transfer for a 6-month period along with a once-off SMS at the beginning of the trial, encouraging them to access routine public sector healthcare services at their nearest government clinic. Cash will be transferred to control arm participants in the same manner as intervention arm participants.

### Qualitative study

Ten caregivers from each arm will be purposively sampled to complete semi-structured in-depth interviews (IDIs) at the start and end of the trial to better understand the lived experiences of caregivers pre- versus post-COVID- 19, perceptions of the intervention and control, and how the intervention may have shaped caregiver wellbeing [28]. Interviews will be guided by an intersectional framework to identify differing experiences of caregivers based on varying positionalities including household socio-economic status, age, ability, and HIV status, and how prior adverse events that may reoccur intersect to impact wellbeing and possible methods through which the intervention works to shape wellbeing. IDIs will be conducted by an experienced qualitative research assistant from the CWeL pilot trial, at baseline and endline, in participant’s preferred language. All interviews will be audio-recorded and based on a semi-structured interview guide that will feature open- and close-ended questions. Key themes from the qualitative data will be routinely shared with investigators for reflection and suggestions on further areas to probe. Refer to Fig. 1 for a visual representation of the study design.

### Economic evaluation

An economic evaluation will be conducted alongside the trial using a micro-costing approach to assess the financial and economic costs of the project from a societal perspective [29]. Cost estimates of staff time, design, training, supervision, workshop sessions, and cash transfers will be conducted in detail. Costs will be treated as either fixed or variable costs. Fixed costs will mainly consist of all study startup costs for vehicles, furniture, and equipment. Variable costs will include (1) consultation costs for developing and finalizing the training manual, (2) operational staffing for running workshops and cash transfers (3), utilities (e.g., rent, water, and electricity), and (4) supplies used in the workshops. Participants’ indirect costs for attending the workshops will also be estimated. These will include transport costs and carer costs. All other inputs will be considered as overheads and calculated using a step-down approach. Cost estimates will be obtained from the project’s financial documents and a time-and-motion study will be conducted to accurately value staffing time spent delivering the workshops.

### Primary outcomes

#### Feasibility measures

This study trial has five feasibility measures:

- Pilot CRT consent rate: The proportion of eligible caregivers approached who consent to participate in the trial. For this trial, a successful consent rate will be defined as one greater than 80%.
- Pilot CRT overall retention rate: The proportion of participants that successfully complete both baseline and endline interviews in the intervention versus the control arm. For this trial, a successful retention rate will be defined as one greater than 85%.
- Intervention arm workshops retention rate: The proportion of participants that successfully attend all workshops in the intervention clusters. For this trial, a successful retention rate will be defined as one greater than 80% compliant.
- Cash transfer protocol adherence: The proportion of participants that successfully receive their cash transfer via the ABSA cash send system in the intervention versus control arm. Successful protocol adherence will be defined as greater than 90% compliance.
- Staff perceptions on project implementation: Perceived barriers and facilitators to study activities from the perspectives of field staff and facilitators (including recruitment, baseline and endline questionnaire administration, intervention and control arm delivery, retention) and strategies for improvement.

#### Potential clinical outcomes for a future trial

In addition to the feasibility measures, this study has three potential primary clinical outcomes: psychological wellbeing, depressive symptoms, and IPV. Depending on the nature of a future grant call and our pilot trial results, including our finalized theory of change model, we will select the most relevant outcome for the future larger trial.

- Psychological wellbeing: assessed based on the percentage change in caregiver wellbeing scores between baseline and endline in the intervention versus control arms. This will be measured using the Mental Health Continuum Short Form [30], a 14-item scale probing subjective, psychological, and social wellbeing (e.g., “In the past month, how often did you feel that you belonged to a community like a social group or your neighborhood”) [31], and the CarerQol- 7D scale, a two-part assessment of the negative and positive effects of caregiving, it includes items such as “I have no/some/a lot of fulfillment from carrying out my care tasks” [32].
- Depressive symptoms: Assessed based on the percentage change in depressive symptom scores between baseline and endline in the intervention versus control arms. Depressive symptoms will be identified using the CES-D- 10 scale, which consists of 10 items (e.g., “During the past week, I was bothered by things that usually don’t bother me”) that assess an individual during the past week [33]. A cut-off score of 12 or higher is deemed optimal to correctly classify individuals as having probable depression for a South African sample [34].
- IPV: Assessed based on the difference in mean scores at endline between the intervention and control arms. Emotional, physical, and sexual IPV will be measured using the WHO’s Violence Against Women scale, which has been extensively used in South Africa [35]. The scale will be summed to create a score of severity of IPV.

#### Secondary outcomes

The secondary outcomes of this trial include: gender attitudes, examined using the 10-item Gender Role’s Belief scale to measure individual perceptions of gender roles [36]; Gender Equality Index which examines self-perceived gender equality in intimate relationships [37]; earnings in the past month, using the mean log of income generated from paid work at baseline compared to endline in the intervention versus control arm; economic empowerment scales that address financial self-efficacy, savings, control over assets, and economic coercion [35]; acceptability of the intervention package and delivery in the intervention versus control arm; total provider cost of intervention delivery; total direct and indirect costs associated with caregiving; and the average cost per participant with an increase in wellbeing score in each arm.

See Additional file 5: Table S1 for a detailed summary of primary clinical outcomes and secondary outcomes.

#### Sample size justification

The chosen sample size of 240 participants was informed by findings from a related economic incentive trial with caregivers of CALHIV [16]. This sample size will enable us to estimate retention of 85% (with *n* = 36 participants or less declining participation or lost to follow-up) to within a 5% margin of error with 95% confidence [16]. In our previous pilot trial, we recruited caregivers (regardless of COVID- 19 SRD grant status) over a 2-month period with a retention rate of 85% at follow-up [16]. This suggests that a sample size of *n* = 240 potentially eligible caregivers for this trial could be recruited and retained within timeframe. Moreover, 10 clusters per arm are within the trial budget.

### Data management

Data will be managed at the SAMRC project office according to the trial’s detailed data collection and quality assurance standard operating procedure. Survey data exports, audio files, and transcripts as well as costing workbooks will be stored in a password-protected project folder on the SAMRC electronic storage drive. The final electronic datasets, codebooks, Stata do files, and questionnaires will be stored on the SAMRC online microdata repository. Upon completion of the project, all data and devices will remain stored in a locked cupboard at the SAMRC-Durban main office, with hard copies of the study forms stored for up to 15 years.

### Statistical analysis

All statistical analysis will be performed using Stata V18.0 [38]. Baseline participant characteristics will be presented using descriptive statistics, including means, medians, and interquartile ranges. Feasibility outcomes will be analyzed using simple descriptive statistics (proportions) and results compared by arm. Linear mixed-effects models will be used for endline scores, adjusting for baseline scores and clustering. To account for the small number of clusters, the Satterthwaite correction will be applied to adjust the degrees of freedom in the linear mixed-effects models [39]. For all binary outcomes, depending on the prevalence of the outcome, we will use mixed effects logistic regression for prevalence less than or equal to 10% and log-binomial or robust Poisson regression when the outcome is common. The number of participants who completed baseline and endline interviews and attendance will be ascertained at each workshop to determine the retention rate.

For the economic evaluation, a breakdown of the total financial and economic costs for key cost categories will be presented to assess the feasibility of the trial, stratified by arm. The average cost per participant will be presented by the outcome. Costs will be presented in 2023 ZAR and USD and adjusted for inflation.

For the qualitative study, electronic audio files will be transcribed and verified by a study team member. A codebook will be developed and transcripts will be coded, with codes grouped into categories and, in turn, themes [40]. An intersectional analysis of the experiences of caregivers will be conducted by heeding stories of the ways intersecting identities shaped experiences during the pandemic [41]. Qualitative data will be used to illuminate preliminary patterns identified in the quantitative data and probe feasibility measures for the trial.

The team of investigators will be consulted to assess whether the hypothesized theory of change [27] was supported by the quantitative and qualitative data and to determine staff perceptions on project implementation; this will allow for the theory of change to be refined and strengthened and to assess the feasibility of the trial (see Table 1 for the study timeline).

**Table 1:** Study timeline

	**Months 1–3**	**Months 4–6**	**Months 7–9**	**Months 10–12**	**Months 13–15**	**Months 16–18**	**Months 19–21**	**Months 22–24**
**Intervention co-development**								
Workshop planning	X							
Workshops (× 3)		X						
Pre-test of intervention		X						
**Pilot CRT**								
Recruitment, baseline interview, follow-up interview (staggered approach by cluster)			X	X	X	X	X	
Intervention Delivery								
Control Delivery			X	X	X	X	X	
**Qualitative study**								
Baseline and endline IDIs			X	X	X	X	X	
**Economic evaluation**								
Cost interviews at baseline and endline			X	X	X	X	X	
**Dissemination**								
Workshops (× 2)								X
Conference presentations								X

## Discussion

Several negative outcomes have been associated with informal caregiving of CALHIV, including depression, IPV, and financial insecurity. These negative outcomes compound with social inequities to impact both caregivers’ own wellbeing and their ability to provide care. Consequently, the aim of this study is to co-develop, test the feasibility, and assess the preliminary effectiveness and cost-effectiveness of a combined unconditional cash transfer and gender transformative economic empowerment intervention on the psychological wellbeing, gender equality, and economic outcomes of caregivers of CALHIV in KwaZulu-Natal, South Africa. The limited access to psycho-social support services and high unemployment during COVID- 19 have intensified the pressures placed on already burdened caregivers of CALHIV [42, 43]. Previous studies have identified the positive synergistic effect of supportive interventions on the relationship between informal caregivers and patients, improving the emotional and mental health of both parties [44]. Further, gender transformative interventions that support women have been found to reduce experiences of IPV and gender inequities by transforming underlying social systems and creating lasting change [19]. Consequently, by incorporating a gender-transformative component, this intervention may improve caregiver wellbeing by helping dismantle the social norms and systems that underlie the gender inequalities caregivers frequently experience. Additionally, economic livelihood strategies that economically empower individuals through improved access to resources have been found to improve livelihoods by increasing independence and social status and enabling women to exercise choice [15]. As such, this intervention has the capacity to provide caregivers with financial assistance that can contribute towards addressing their personal, caregiver, and household financial needs and concerns.

### Strengths

This study builds on previously conducted trials by incorporating themes such as gender equality and IPV, with the goal of evaluating the feasibility, effectiveness, cost-effectiveness, and acceptability of interventions aiming to improve the wellbeing and address the challenges of caregivers of CALHIV post-COVID- 19. In addition, this study employs a human-centered research design that centers the knowledge, experiences, and needs of community members through the co-development of intervention materials with an existing CAB, allowing power differentials inherent to the research-participant relationship to be addressed [45]. Further, the economic evaluation fills a notable knowledge gap regarding the cost-effectiveness of wellbeing-related interventions in South Africa. Finally, through the intervention co-development phase and incorporation of a CAB, this study acknowledges power dynamics inherent in the researcher-participant relationship to strengthen this participatory approach and facilitate the creation of a quality intervention that is sustainable, empowering, and builds capacity [46].

### Ethical considerations

Voluntary written informed consent will be obtained from participants prior to participation in the participant’s preferred language. Ethics approval for this trial has been granted from the SAMRC Human Research Ethics Committee (EC006 - 2/2023), the KwaZulu-Natal Department of Health (KZN DoH) (KZ_202304_010), and Simon Fraser University (30,001,806), a key collaborating institution. All participants will be reimbursed at least R150 for each physical interaction with the study team (i.e., baseline and follow-up surveys, workshops, and qualitative interviews). This amount will be for their time (i.e., R50), transportation (R50), lunch (R50), and carer costs (If applicable, will be treated on a case-by-case basis).

The trial is subject to the following ethical considerations. Notably, given the sensitive nature of certain questions or modules, there is the potential risk of participant discomfort or distress. To minimize this risk, female fieldworkers with a background in GBV research and HIV counseling have been hired. Additionally, fieldworkers have been trained to identify early signs of discomfort or distress, and counsel and link participants to appropriate care accordingly. Further, there is the potential to identify victims and perpetrators of abuse throughout the screening and workshop procedures. The study team will adhere to reporting requirements of the Children’s Act of 2005—Sect. 110 in the case of reported or suspected child abuse [47]. Additionally, a referral system will be implemented to ensure vulnerable groups or participants who have disclosed experiences of GBV or abuse are linked to local health care and social services that can adequately respond. Informed consent forms will highlight the procedures, with fieldworkers assuring participants that their confidentiality will be respected but managing expectations.

### Limitations

The trial is subject to the following limitations: (1) the sample is restricted to female caregivers accessing HIV clinics who are eligible for the COVID- 19 SRD grant, potentially limiting the generalizability of our findings. (2) There is the potential for social desirability bias to be present due to the sensitivity of the topics proved during the questionnaires and workshops. However, the trial attempts to minimize this bias by building rapport with participants throughout the process and prioritizing the creation of a safe space for participants to share their stories. (3) Participants have full autonomy on how they use the cash transfer each month meaning there is no guarantee that participants will utilize their cash transfer to improve their economic livelihoods through savings or other income-generating activities. (4) The sample size power calculation does not consider IPV despite it being a primary outcome. However, previous IPV studies have demonstrated that adequate time and sample are needed to be powered to make an impact on IPV [35, 48–50]. As such, findings from this trial will inform future studies that could address this limitation by implementing this intervention on a larger scale.

### Anticipated use of results

Ultimately, the results from this trial will contribute to our knowledge of the association between psychological wellbeing and gender inequities of caregivers in South Africa and potential strategies to enhance wellbeing and gender inequities. Further, this trial will improve our understanding of the challenges of implementing a long-term economic incentive to mitigate the negative impacts of caregiving in this context and potential factors that may impact the effectiveness of an intervention of this nature. Finally, this preliminary trial has the potential to inform a larger confirmatory trial which could be useful to policy makers when considering strategies to sustain the COVID- 19 SRD grant, post-pandemic recovery efforts, and social assistance programs for caregivers of CALHIV and others disproportionately burdened by compounding health and social crises.

### Trial status

Enrollment of clusters complete. Seven clusters have completed the intervention economic empowerment workshops, and three clusters are in progress. Baseline quantitative complete (intervention: *n* = 149, control: *n* = 124) and qualitative interviews (intervention: *n* = 10, control: *n* = 10 complete. Cash transfers are still ongoing, all clusters have received at least 1 payment.

## Supplementary Information

Additional file 1: Appendix A—SPIRIT Checklist. Checklist of recommended items to address in a clinical trial protocol.

Additional file 2: Appendix C—SRD Grant Eligibility Criteria. Eligibility criteria for South Africa’s COVID- 19 SRD grant.

Additional file 3: Appendix B—Baseline and endline questionnaires. Baseline and endline questionnaires utilized during pilot CRT.

Additional file 4: Appendix D – Proposed Theories of Change. Proposed Theories of Change for CWeL and SSCF trials.

Additional file 5: Table S1. Primary and secondary outcomes of the caregiver wellness + study. Detailed summary of primary clinical and secondary outcomes.

## Data Availability

The data and materials from this trial are managed by the SAMRC online microdata repository and available on request. Access to the dataset will be limited to researchers whose research topic or analysis places are compatible, with consent and assent agreements from participants.

## Abbreviations

CAB: Caregiver Advisory Board
CALHIV: Children and adolescents living with HIV
CONSORT: Consolidated Standards for Reporting Trials
CRT: Cluster randomized trial
GBV: Gender-based violence
IDI: In-depth interviews
IPV: Intimate partner violence
KZN DoH: KwaZulu-Natal Department of Health
SAMRC: South African Medical Research Council
SMS: Short text messaging
SPIRIT: Standard Protocol Items Recommendations for Interventional Trials
SRD: Social Relief of Distress
SSCF: Stepping Stones and Creating Futures

## References

[1] UNICEF. HIV statistics - global and regional trends 2023 [updated 2023/10/20/07:42:36]. Available from: https://data.unicef.org/topic/hivaids/global-regional-trends/.

[2] Shenderovich Y, Boyes M, Esposti MD, Casale M, Toska E, Roberts KJ, et al. Relationships with caregivers and mental health outcomes among adolescents living with HIV: a prospective cohort study in South Africa. BMC Public Health. 2021;21(1):172. 33472607 10.1186/s12889-020-10147-z PMC7816135

[3] Osafo J, Knizek BL, Mugisha J, Kinyanda E. The experiences of caregivers of children living with HIV and AIDS in Uganda: a qualitative study. Glob Health. 2017;13(1):72. 10.1186/s12992-017-0294-9 PMC5596491 28899415

[4] Magaña I, Martínez P, Loyola M-S. Health outcomes of unpaid caregivers in low- and middle-income countries: a systematic review and meta-analysis. J Clin Nurs. 2020;29(21–22):3950–65. 32786156 10.1111/jocn.15450

[5] Currie J. Child health as human capital. Health Econ. 2020;29(4):452–63. 31965679 10.1002/hec.3995

[6] Govindasamy D, Ferrari G, Maruping K, Bodzo P, Mathews C, Seeley J. A qualitative enquiry into the meaning and experiences of wellbeing among young people living with and without HIV in KwaZulu-Natal. South Africa Social Science & Medicine. 2020;258:113103. 32535474 10.1016/j.socscimed.2020.113103

[7] Dang HAH, Viet Nguyen C. Gender inequality during the COVID-19 pandemic: income, expenditure, savings, and job loss. World Development. 2021;140:105296. 34548740 10.1016/j.worlddev.2020.105296 PMC8446715

[8] Blundell R, Costa Dias M, Joyce R, Xu X. COVID-19 and inequalities. Fisc Stud. 2020;41(2):291–319. 32836542 10.1111/1475-5890.12232 PMC7362053

[9] South African Department of Social Department. The rapid assessment of the implementation and utilisation of the special COVID-19 SRD grant. Pretoria: South African Government, Department of Social Development, 2021.

[10] Devereux S. Social protection responses to COVID-19 in Africa. Global Social Policy. 2021;21(3):421–47.

[11] Goldman M, Bassier I, Budlender J, Mzankomo L, Woolard I, Leibbrandt MV. Simulation of options to replace the special COVID-19 Social Relief of Distress grant and close the poverty gap at the food poverty line. WIDER Working Paper, 2021 9292671057.

[12] United Nations Development Programme. Update on UNDP's Socio-economic Response Beyond Recovery: Towards 2030. 2021.

[13] Vyas S, Watts C. How does economic empowerment affect women’s risk of intimate partner violence in low and middle income countries? A systematic review of published evidence. J Int Dev. 2009;21(5):577–602.

[14] World Health Organization. Community-based rehabilitation: CBR guidelines 2023 [updated 2023/10/20/09:22:31]. Available from: https://www.who.int/publications-detail-redirect/9789241548052. 26290927

[15] Laszlo S, Grantham K, Oskay E, Zhang T. Grappling with the challenges of measuring women’s economic empowerment in intrahousehold settings. World Development. 2020;132:104959.

[16] Carries S, Mkhwanazi Z, Sigwadhi L, Moshabela M, Nyirenda M, Goudge J, et al. An economic incentive package to support the wellbeing of caregivers of adolescents living with HIV during the COVID-19 pandemic in South Africa: a feasibility study protocol for a pilot randomised trial. Pilot Feasibility Stud. 2023;9(1):3. 36624520 10.1186/s40814-023-01237-x PMC9827020

[17] Gibbs A, Washington L, Abdelatif N, Chirwa E, Willan S, Shai N, et al. Stepping stones and creating futures intervention to prevent intimate partner violence among young people: cluster randomized controlled trial. J Adolesc Health. 2020;66(3):323–35. 31784410 10.1016/j.jadohealth.2019.10.004

[18] Clancy K, Ink S, Do C, Melesse M, Nijhawan T, Njuki J, et al. Transforming gender relations: insights from IDRC research by IDRC 2019 [updated 2019/05/29/ 2023/10/20/09:28:09]. Available from: https://issuu.com/idrc_crdi/docs/wd_13_000_gender_e-file_en.

[19] Hillenbrand E, Karim N, Mohanraj P, Wu D. Measuring gender-transformative change: A review of literature and promising practices. 2015.

[20] Dworkin SL, Treves-Kagan S, Lippman SA. Gender-transformative interventions to reduce HIV risks and violence with heterosexually-active men: a review of the global evidence. AIDS Behav. 2013;17(9):2845–63. 23934267 10.1007/s10461-013-0565-2

[21] Thabane L, Lancaster G. A guide to the reporting of protocols of pilot and feasibility trials. Springer. 2019. 10.1186/s40814-019-0423-8 PMC6393983 30858987

[22] SPIRIT. Guidance for clinical trial protocols 2023 [updated 2023/12/25/21:54:50]. Available from: https://www.spirit-statement.org/.

[23] Eldridge SM, Chan CL, Campbell MJ, Bond CM, Hopewell S, Thabane L, et al. CONSORT 2010 statement: extension to randomised pilot and feasibility trials. BMJ. 2016;355:i5239. 27777223 10.1136/bmj.i5239 PMC5076380

[24] Harris PA, Taylor R, Minor BL, Elliott V, Fernandez M, O’Neal L, et al. The REDCap consortium: building an international community of software partners. Journal of Biomedical Informatics. 2019;95:103208. 31078660 10.1016/j.jbi.2019.103208 PMC7254481

[25] Harris PA, Taylor R, Thielke R, Payne J, Gonzalez N, Conde JG. Research electronic data capture (REDCap) – a metadata-driven methodology and workflow process for providing translational research informatics support. J Biomed Inform. 2009;42(2):388–91. 10.1016/j.jbi.2008.08.010 PMC2700030 18929686

[26] Parker AR. Another point of view: a manual on gender analysis training for grassroots workers; training manual. 1993

[27] Gibbs A, Washington L, Willan S, Ntini N, Khumalo T, Mbatha N, et al. The stepping stones and creating futures intervention to prevent intimate partner violence and HIV-risk behaviours in Durban, South Africa: study protocol for a cluster randomized control trial, and baseline characteristics. BMC Public Health. 2017;17(1):336. 28427380 10.1186/s12889-017-4223-x PMC5397780

[28] Moore GF, Audrey S, Barker M, Bond L, Bonell C, Hardeman W, et al. Process evaluation of complex interventions: Medical Research Council guidance. BMJ. 2015;350. 10.1136/bmj.h1258 PMC4366184 25791983

[29] Drummond MF, Sculpher MJ, Claxton K, Stoddart GL, Torrance GW. Methods for the economic evaluation of health care programmes: Oxford University Press. 2015

[30] Keyes CLM. Social well-being. Social Psychology Quarterly. 1998;61(2):121–40.

[31] Keyes CLM, Wissing M, Potgieter J, Temane M, Kruger A, van Rooy S. Evaluation of the mental health continuum - short form (MHC-SF) in Setswana-speaking South Africans. Clin Psychol Psychother. 2008;15:181–92. 19115439 10.1002/cpp.572

[32] Brouwer WBF, van Exel NJA, van Gorp B, Redekop WK. The CarerQol instrument: a new instrument to measure care-related quality of life of informal caregivers for use in economic evaluations. Qual Life Res. 2006;15(6):1005–21. 16900281 10.1007/s11136-005-5994-6

[33] Radloff LS. The CES-D Scale: a self-report depression scale for research in the general population. Appl Psychol Meas. 1977;1(3):385–401.

[34] Baron E, Davies T, Lund C. Validation of the 10-item Centre for Epidemiological Studies Depression Scale (CES-D-10) in Zulu, Xhosa and Afrikaans populations in South Africa. BMC Psychiatry. 2017;17:1–4. 28068955 10.1186/s12888-016-1178-x PMC5223549

[35] Jewkes RK, Dunkle K, Nduna M, Shai N. Intimate partner violence, relationship power inequity, and incidence of HIV infection in young women in South Africa: a cohort study. The Lancet. 2010;376(9734):41–8. 10.1016/S0140-6736(10)60548-X 20557928

[36] Brown MJ, Gladstone N. Development of a Short version of the gender role beliefs scale. International Journal of Psychology and Behavioral Sciences. 2012;2(5):154–8.

[37] Sörlin A, Lindholm L, Ng N, Öhman A. Gender equality in couples and self-rated health - A survey study evaluating measurements of gender equality and its impact on health. International Journal for Equity in Health. 2011;10:1-11. 10.1186/1475-9276-10-37 PMC3167759 21871087

[38] StataCorp LLC. StataCorp. Stata Statistical Software: Release 18. 2023.

[39] Leyrat C, Morgan KE, Leurent B, Kahan BC. Cluster randomized trials with a small number of clusters: which analyses should be used? Int J Epidemiol. 2017;47(1):321–31. 10.1093/ije/dyx169 29025158

[40] Gale NK, Heath G, Cameron E, Rashid S, Redwood S. Using the framework method for the analysis of qualitative data in multi-disciplinary health research. BMC Med Res Methodol. 2013;13(1):117. 24047204 10.1186/1471-2288-13-117 PMC3848812

[41] Abrams JA, Tabaac A, Jung S, Else-Quest NM. Considerations for employing intersectionality in qualitative health research. Soc Sci Med. 2020;258:113138. 32574889 10.1016/j.socscimed.2020.113138 PMC7363589

[42] Cluver L, Lachman JM, Sherr L, Wessels I, Krug E, Rakotomalala S, et al. Parenting in a time of COVID-19. Lancet. 2020;395(10231):e64. 32220657 10.1016/S0140-6736(20)30736-4 PMC7146667

[43] Panda PK, Gupta J, Chowdhury SR, Kumar R, Meena AK, Madaan P, et al. Psychological and behavioral impact of lockdown and quarantine measures for COVID-19 pandemic on children, adolescents and caregivers: a systematic review and meta-analysis. Journal of Tropical Pediatrics. 2021;67(1):fmaa122. 10.1093/tropej/fmaa122 PMC7798512 33367907

[44] Schnall R, Hirshfield S, Liu J, Siegel K, Gradilla M. Characteristics of persons living with HIV who have informal caregivers in the cART age of the epidemic. J Assoc Nurses AIDS Care. 2018;29(2):152–62. 28941571 10.1016/j.jana.2017.08.008 PMC5816723

[45] Macaulay AC, Commanda LE, Freeman WL, Gibson N, McCabe ML, Robbins CM, et al. Participatory research maximises community and lay involvement. BMJ. 1999;319(7212):774–8. 10488012 10.1136/bmj.319.7212.774 PMC1116604

[46] Jagosh J, Macaulay AC, Pluye P, Salsberg J, Bush PL, Henderson J, et al. Uncovering the benefits of participatory research: implications of a realist review for health research and practice. Milbank Q. 2012;90(2):311–46. 22709390 10.1111/j.1468-0009.2012.00665.x PMC3460206

[47] Government SA. Children's Act 38 of 2005 2005 [updated 2023/12/17/06:57:19]. Available from: https://www.gov.za/documents/childrens-act.

[48] Jewkes R. Intimate partner violence: causes and prevention. Lancet. 2002;359(9315):1423–9. 11978358 10.1016/S0140-6736(02)08357-5

[49] Jewkes R, Gibbs A, Jama-Shai N, Willan S, Misselhorn A, Mushinga M, et al. Stepping Stones and Creating Futures intervention: shortened interrupted time series evaluation of a behavioural and structural health promotion and violence prevention intervention for young people in informal settlements in Durban, South Africa. BMC Public Health. 2014;14(1):1325. 25544716 10.1186/1471-2458-14-1325 PMC4320600

[50] Jewkes R, Willan S, Heise L, Washington L, Shai N, Kerr-Wilson A, et al. Elements of the Design and Implementation of Interventions to Prevent Violence against Women and Girls associated with success: reflections from the what works to prevent violence against women and girls? Global Programme. Int J Environ Res Public Health. 2021;18(22):12129. 34831885 10.3390/ijerph182212129 PMC8621962

