# Supplementary material for: A cash transfer plus gender transformative economic empowerment intervention seeking to improve the wellbeing of caregivers of children and adolescents living with HIV in South Africa: a feasibility study protocol for a pilot cluster randomized trial
Source: Pilot Feasibility Stud. 2025 Apr 23;11:52. doi: 10.1186/s40814-025-01643-3 (PMC12020082; doi:10.1186/s40814-025-01643-3)
Supplement: Supplementary file 2 — Additional file 2: Appendix C—SRD Grant Eligibility Criteria. Eligibility criteria for South Africa’s COVID- 19 SRD grant. [file 40814_2025_1643_MOESM2_ESM.pdf]

Appendix B

SRD Grant Eligibility Criteria

To be eligible for the SRD grant, individuals must be:

- Citizens and residents of South Africa.
- Between the ages of 18 and 58; unemployed.
- Not receiving any income, social grant
- Unemployment insurance benefits (and does not qualify), stipends from the National Financial Aid Scheme, other government COVID-19 response support.
- Not residing in a government funded or subsidised institution.
