# Supplementary material for: A cash transfer plus gender transformative economic empowerment intervention seeking to improve the wellbeing of caregivers of children and adolescents living with HIV in South Africa: a feasibility study protocol for a pilot cluster randomized trial
Source: Pilot Feasibility Stud. 2025 Apr 23;11:52. doi: 10.1186/s40814-025-01643-3 (PMC12020082; doi:10.1186/s40814-025-01643-3)
Supplement: Supplementary file 3 — Additional file 3: Appendix B—Baseline and endline questionnaires. Baseline and endline questionnaires utilized during pilot CRT. [file 40814_2025_1643_MOESM3_ESM.pdf]

Appendix C
Baseline and endline questionnaires

SECTION A: DEMOGRAPHICS

Question	Response
1. How old are you today?	Age: ___________<br><br>☐ Don't know<br>☐ Refused
2. What is your date of birth? (dd MM yyyy)	________________<br>dd/ mm/ yyyy
3. What is your assigned sex at birth?	☐ Female<br>☐ Male<br>☐ Intersex<br>☐ Other (*Specify below*)<br>_______________
4. What gender do you identify with currently?	☐ Girl/Woman<br>☐ Boy/Man<br>☐ Non-binary or gender fluid<br>☐ Agender/Genderless<br>☐ Transgender (Woman)<br>☐ Transgender (Man)<br>☐ Other (*Specify below*)<br>________________
5. Who do you feel sexually attracted to?	☐ Heterosexual/Straight<br>☐ Lesbian or Gay<br>☐ Bisexual<br>☐ Asexual<br>☐ MSM (Men who have sex with men)<br>☐ WSW (Women who have sex with women)<br>☐ Pansexual<br>☐ Queer<br>☐ Undecided/Questioning<br>☐ Other (*Specify below*)<br>________________
6. What is your race?	☐ Black/African<br>☐ Indian/Asian<br>☐ Coloured<br>☐ White<br>☐ Other (*Specify below*)

	________________<br><br>☐Refused
7. What is the highest grade or standard they you have passed?	________________
8. What is the highest tertiary qualification(s) you have passed like diplomas, certificates or degrees?	☐ None<br>☐ Certificate<br>☐ Diploma<br>☐ Undergraduate degree<br>☐ Postgraduate degree<br>☐ Not applicable
9. Do you currently have a partner(s)?	☐ Yes<br>☐ No
10. (If yes to Q9) What type of relationship are you in?	☐ Legally married<br>☐ Traditionally married<br>☐ Partner recently died<br>☐ Engaged to be married<br>☐ Dating short-term (<6 months)<br>☐ Dating long term (>6 months)<br>☐ Casual
11. How many partners have you had in the past 6 months (including current partner(s))?	☐ 0<br>☐ 1<br>☐ 2<br>☐ >2
12. If you have a partner or had a partner(s) in the last 6 months, what gender do they identify with?	☐ Girl/Woman<br>☐ Boy/Man<br>☐Non-binary or gender fluid<br>☐Agender/Genderless<br>☐Transgender (Woman)<br>☐ Transgender (Man)<br>☐ Other (*Specify below*)<br><br>________________
13. If you have a partner or had a partner(s) in the last 6 months, what was their sexual orientation?	☐ Heterosexual/Straight<br>☐ Lesbian or Gay<br>☐ Bisexual<br>☐ Asexual<br>☐ MSM (Men who have sex with men)<br>☐ WSW (Women who have sex with women)<br>☐ Pansexual<br>☐ Queer<br>☐ Undecided/Questioning<br>☐ Other (*Specify below*)<br><br>________________

14. If you have or had a partner in the last 6 months, how long is/was the relationship(s)?	________________	
15. If you have a partner or are married, do they currently live with you?	☐ Yes<br>☐ No	
16. What type of house/dwelling do you live in?	☐ House/flat/RDP house<br>☐ Informal house (e.g. shack)<br>☐ Traditional house (e.g. mud house)<br>☐ Don't know	
17. Do you have piped or tap water inside your dwelling or hour or in your yard?	☐ Yes<br>☐ No	
18. Does your dwelling or house have access to electricity?	☐ Yes<br>☐ No	
19. Tell me about any health problems you are currently dealing with?	☐ TB<br>☐ Asthma<br>☐ Diabetes<br>☐ Hypertension<br>☐ HIV<br>☐ HIV – On ART<br>☐ HIV – Not on ART<br>☐ Cancer<br>☐ COVID-19<br>☐ Arthritis	☐ Kidney disease<br>☐ Heart condition<br>☐ Lung condition<br>☐ Skin disorder<br>☐ Mental health related (e.g. anxiety, depression, or bipolar).<br>☐ Other<br>☐ Refused<br>☐ None
19a. Please specify other health problems	________________________________	
20. Do you have any of the following disabilities?	☐ Hearing impairment<br>☐ Mental disability<br>☐ Speech impediment<br>☐ Unable to walk properly<br>☐ Visual impairment/ Blindness	☐ Wheelchair-bound<br>☐ Other<br>☐ Refused<br>☐ None
21. Specify other medical conditions/disability	________________________________	
22. I would like to now focus on the number of people that are living in your household (i.e. people sleeping under the same roof or eating from the same pot)		
i. How many people in your household are 5 years old or younger?	________________	
ii. How many people in your household are 6 to 18 years old?	________________	

iii.	How many people in your household are 19 to 60 years old?	________________
iv.	How many people in your household are older than 60 years?	________________
v.	Total number of people in your household?	________________

SECTION B: HEALTH RELATED QUALITY OF LIFE

EQ-5D-5L

MOBILITY
- ☐ I have no problems in walking about
- ☐ I have slight problems in walking about
- ☐ I have moderate problems in walking about
- ☐ I have severe problems in walking about
- ☐ I am unable to walk about

SELF-CARE
- ☐ I have no problems washing or dressing myself
- ☐ I have slight problems washing or dressing myself
- ☐ I have moderate problems washing or dressing myself
- ☐ I have severe problems washing or dressing myself
- ☐ I am unable to wash or dress myself

UNUSUAL ACTIVITIES (e.g. work, study, housework, family or leisure activities)
- ☐ I have no problems doing my usual activities
- ☐ I have slight problems doing my usual activities
- ☐ I have moderate problems doing my usual activities
- ☐ I have severe problems doing my usual activities
- ☐ I am unable to do my usual activities

PAIN / DISCOMFORT
- ☐ I have no pain or discomfort
- ☐ I have slight pain or discomfort
- ☐ I have moderate pain or discomfort
- ☐ I have severe pain or discomfort
- ☐ I have extreme pain or discomfort

ANXIETY / DEPRESSION
- ☐ I am not anxious or depressed
- ☐ I am slightly anxious or depressed
- ☐ I am moderately anxious or depressed
- ☐ I am severely anxious or depressed
- ☐ I am extremely anxious or depressed

WE WOULD LIKE TO KNOW HOW GOOD OR BAD YOUR HEALTH IS **TODAY**

- This scale is numbered from 0 to 100.
- 100 means the best health you can imagine. 0 means the worst health you can imagine.
- Mark an X on the scale to indicate how your health is **TODAY**
- Now, please write the number you marked on the scale in

YOUR HEALTH TODAY = ☐

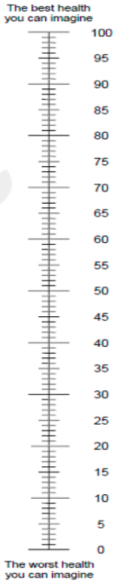

SECTION C: PSYCHOLOGICAL WELLBEING

Mental Health Continuum Short Form (MHC-SF)

**The following questions are about how you have been feeling during past month. Place a check mark in the box that best represents how often you have experienced or felt the following:**						
**During the past month how often did you feel…**	**Never**	**Once or twice**	**About once a week**	**About 2 to 3 times a week**	**Almost every day**	**Every day**
1. happy						
2. interested in life						
3. satisfied						
4. that you had something important to contribute to society						
5. that you belonged to a community (like a social group, or your neighbourhood)						

6. that our society is becoming a better place for people like you						
7. that people are basically good						
8. that the way our society works makes sense to you						
9. that you liked most parts of your personality						
10. good at managing the responsibilities of your daily life						
11. that you had warm and trusting relationships with others						
12. that you had experiences that challenged you to grow and become a better person						
13. confident to think or express your own ideas and opinions						
14. that your life has a sense of direction or meaning to it						

CarerQol-7D

We would like to form an impression of your caregiving situation.
Please tick a box to indicate which description best fits your caregiving situation at the moment.

Please tick only one box per description: 'no', 'some' or 'a lot of'.

	no	some	a lot of	
1. I have				fulfilment from carrying out my care tasks.
2. I have				relational problems with the care receiver (*e.g., he/she is very demanding or he/she behaves differently; we have communication problems*).
3. I have				problems with my own mental health (*e.g., stress, fear, gloominess, depression, concern about the future*).
4. I have				problems combining my care tasks with my daily activities (*e.g., household activities, work, study, family and leisure activities*).
5. I have				financial problems because of my care tasks.
6. I have				support with carrying out my care tasks, when I need it (*e.g., from family, friends, neighbours, acquaintances*).
7. I have				problems with my own physical health (*e.g., more often sick, tiredness, physical stress*).

CarerQol-VAS

How happy do you feel at the moment?

Please place a mark on the scale below that indicates how happy you feel at the moment.

completely unhappy — completely happy

0 1 2 3 4 5 6 7 8 9 10

SECTION D: DEPRESSIVE SYMPTOMS

Centre for Epidemiological Studies Depression Scale (CES-D-10)

During the Past Week

	Rarely or none of the time (less than 1 day)	Some or a little of the time (1-2 days)	Occasionally or a moderate amount of time (3-4 days)	Most or all of the time (5-7 days)
1. I was bothered by things that usually don't bother me.	☐	☐	☐	☐
2. I had trouble keeping my mind on what I was doing.	☐	☐	☐	☐
3. I felt depressed.	☐	☐	☐	☐
4. I felt that everything I did was an effort.	☐	☐	☐	☐
5. I felt hopeful about the future.	☐	☐	☐	☐
6. I felt fearful.	☐	☐	☐	☐
7. My sleep was restless.	☐	☐	☐	☐
8. I was happy.	☐	☐	☐	☐
9. I felt lonely.	☐	☐	☐	☐
10. I could not "get going."	☐	☐	☐	☐

SECTION E: INTIMATE PARTNER VIOLENCE (IPV)

WHO's Violence Against Women Scale – Past 12 months

**Psychological/Emotional IPV - Victimisation**					
A	In the past 12 months how many times has a current or previous partner insulted you or made you feel bad about yourself?	NEVER	ONCE	FEW	MANY
B	In the past 12 months how many times has a current or previous partner belittled or humiliated you in front of other people?	1	2	3	4
C	In the past 12 months how many times has a current or previous partner done things to scare or intimidate you on purpose for example, by the way he looked at you, by yelling or smashing things?	1	2	3	4
D	In the past 12 months how many times has a current or previous partner threatened to hurt you?	1	2	3	4
E	In the past 12 months how many times has a current or previous partner hurt people you care about as a way of hurting you, or damaged things of importance to you?	1	2	3	4

**Physical IPV - Victimisation**					
We are interested now in your relationship with your current or previous partner. The following questions relate to things which may have been done by your partner when you did not want them to.					
A	In the past 12 months how many times has a current or previous partner ever slapped you or thrown something at you which could hurt you?	NEVER	ONCE	FEW	MANY
B	In the past 12 months how many times has a current or previous partner ever pushed or shoved you?	1	2	3	4
C	In the past 12 months how many times has a current or previous partner ever hit you with a fist or with something else which could hurt you?	1	2	3	4
D	In the past 12 months, how many times has a current or previous partner ever kicked, dragged, beaten, choked or burnt you?	1	2	3	4
E	In the past 12 months, how many times has a current or previous partner ever threatened to	1	2	3	4

	use or actually used a gun, knife or other weapon against you?				

**Sexual IPV - Victimisation**					
The next few questions are about things you may have done with a current or previous partner.					
A	In the past 12 months, how many times has a current or previous partner ever physically forced you to have sex when you did not want to?	NEVER	ONCE	FEW	MANY
B	In the past 12 months, how many times has your current or previous partner or partner used threats or intimidation to get you to have sex when you did not want to?	1	2	3	4
C	In the past 12 months, how many times has a current or previous partner ever forced you to do something else sexual that did not want to do?	1	2	3	4

**Economic Violence - Victimisation**					
A	In the past 12 months how often did your partner stop you from getting a job, going to work, trading or earning money?	NEVER	ONCE	FEW	MANY
B	In the past 12 months how often did your partner take your earnings against your will?	1	2	3	4
C	In the past 12 months how often did your partner throw you out of the house?	1	2	3	4
D	In the past 12 months how often did your partner lock you in a house or room so you could not leave?	1	2	3	4
E	In the past 12 months how often did your partner spend money on alcohol, tobacco or other things for himself when he knew you did not have enough for essential household expenses?	1	2	3	4

**Psychological/Emotional IPV - Perpetration**					
A	In the past 12 months how many times have you insulted a current or previous partner or made them feel bad about themselves?	NEVER	ONCE	FEW	MANY
B	In the past 12 months how many times have you belittled or humiliated a current or previous partner in front of other people?	1	2	3	4
C	In the past 12 months how many times have you done things to scare or intimidate a current or	1	2	3	4

	previous partner on purpose for example, by the way you looked at them, by yelling or smashing things?				
D	In the past 12 months how many times have you threatened to hurt a current or previous partner?	1	2	3	4
E	In the past 12 months how many times have you hurt people a current or previous partner cares about as a way of hurting them, or damaged things of importance to them?	1	2	3	4

**Physical IPV - Perpetration**					
We are interested now in your relationship with your current or previous partner. The following questions relate to things you may have been done to your partner when they did not want you to.					
A	In the past 12 months how many times have you ever slapped a current or previous partner or thrown something at them which could hurt them?	NEVER	ONCE	FEW	MANY
B	In the past 12 months how many times have you ever pushed or shoved a current or previous partner?	1	2	3	4
C	In the past 12 months how, many times have you ever hit with a fist or something else that could hurt your current or previous partner?	1	2	3	4
D	In the past 12 months, how many times have you ever kicked, dragged, beaten, choked or burnt a current or previous partner?	1	2	3	4
E	In the past 12 months, how many times have you ever threatened to use or actually used a gun, knife or other weapon against a current or previous partner?	1	2	3	4

**Sexual IPV - Perpetration**					
A	In the past 12 months, how many times have you physically forced a current or previous partner to have sex with you when they did not want to?	NEVER	ONCE	FEW	MANY
B	In the past 12 months, how many times have you used threats or intimidation to get your current or previous partner to have sex with you when they did not want to?	1	2	3	4
C	In the past 12 months, how many times have you forced a current or previous partner to do	1	2	3	4

	something else sexual that they did not want to do?				

Economic IPV - Perpetration					
A	In the past 12 months how often did you stop your partner from getting a job, going to work, trading or earning money?	NEVER	ONCE	FEW	MANY
B	In the past 12 months how often did you take your partner's earnings against their will?	1	2	3	4
C	In the past 12 months how often did you throw your partner out of the house?	1	2	3	4
D	In the past 12 months how often did you lock your partner in a house or room so they could not leave?	1	2	3	4
E	In the past 12 months how often did you spend money on alcohol, tobacco or other things for yourself when you knew you did not have enough for essential household expenses?	1	2	3	4

WHO's Violence Against Women Scale – Past 6 months

Psychological/Emotional IPV - Victimisation					
A	In the past 6 months how many times has a current or previous partner insulted you or made you feel bad about yourself?	NEVER	ONCE	FEW	MANY
B	In the past 6 months how many times has a current or previous partner belittled or humiliated you in front of other people?	1	2	3	4
C	In the past 6 months how many times has a current or previous partner done things to scare or intimidate you on purpose for example, by the way he looked at you, by yelling or smashing things?	1	2	3	4
D	In the past 6 months how many times has a current or previous<br>partner threatened to hurt you?	1	2	3	4
E	In the past 6 months how many times has a current or previous partner hurt people you care about as a way of hurting you, or damaged things of importance to you?	1	2	3	4

Physical IPV - Victimisation

We are interested now in your relationship with your current or previous partner. The following questions relate to things which may have been done by your partner when you did not want them to.					
A	In the past 6 months how many times has a current or previous partner ever slapped you or thrown something at you which could hurt you?	NEVER	ONCE	FEW	MANY
B	In the past 6 months how many times has a current or previous partner ever pushed or shoved you?	1	2	3	4
C	In the past 6 months how many times has a current or previous partner ever hit you with a fist or with something else which could hurt you?	1	2	3	4
D	In the past 6 months, how many times has a current or previous partner ever kicked, dragged, beaten, choked or burnt you?	1	2	3	4
E	In the past 6 months, how many times has a current or previous partner ever threatened to use or actually used a gun, knife or other weapon against you?	1	2	3	4

**Sexual IPV - Victimisation**					
The next few questions are about things you may have done with a <u>current or previous partner</u>.					
A	In the past 6 months, how many times has a current or previous partner ever physically forced you to have sex when you did not want to?	NEVER	ONCE	FEW	MANY
B	In the past 6 months, how many times has your current or previous partner or partner used threats or intimidation to get you to have sex when you did not want to?	1	2	3	4
C	In the past 6 months, how many times has a current or previous partner ever forced you to do something else sexual that did not want to do?	1	2	3	4

**Economic Violence - Victimisation**					
A	In the past 6 months how often did your partner stop you from getting a job, going to work, trading or earning money?	NEVER	ONCE	FEW	MANY
B	In the past 6 months how often did your partner take your earnings against your will?	1	2	3	4
C	In the past 6 months how often did your partner throw you out of the house?	1	2	3	4

D	In the past 6 months how often did your partner lock you in a house or room so you could not leave?	1	2	3	4
E	In the past 6 months how often did your partner spend money on alcohol, tobacco or other things for himself when he knew you did not have enough for essential household expenses?	1	2	3	4

**Psychological/Emotional IPV - Perpetration**					
A	In the past 6 months how many times have you insulted a current or previous partner or made them feel bad about themselves?	NEVER	ONCE	FEW	MANY
B	In the past 6 months how many times have you belittled or humiliated a current or previous partner in front of other people?	1	2	3	4
C	In the past 6 months how many times have you done things to scare or intimidate a current or previous partner on purpose for example, by the way you looked at them, by yelling or smashing things?	1	2	3	4
D	In the past 6 months how many times have you threatened to hurt a current or previous partner?	1	2	3	4
E	In the past 6 months how many times have you hurt people a current or previous partner cares about as a way of hurting them, or damaged things of importance to them?	1	2	3	4

**Physical IPV - Perpetration**					
We are interested now in your relationship with your current or previous partner. The following questions relate to things you may have been done to your partner when they did not want you to.					
A	In the past 6 months how many times have you ever slapped a current or previous partner or thrown something at them which could hurt them?	NEVER	ONCE	FEW	MANY
B	In the past 6 months how many times have you ever pushed or shoved a current or previous partner?	1	2	3	4
C	In the past 6 months how, many times have you ever hit with a fist or something else that could hurt your current or previous partner?	1	2	3	4
D	In the past 6 months, how many times have you ever kicked, dragged, beaten, choked or burnt a current or previous partner?	1	2	3	4

E	In the past 6 months, how many times have you ever threatened to use or actually used a gun, knife or other weapon against a current or previous partner?	1	2	3	4

**Sexual IPV - Perpetration**					
A	In the past 6 months, how many times have you physically forced a current or previous partner to have sex with you when they did not want to?	NEVER	ONCE	FEW	MANY
B	In the past 6 months, how many times have you used threats or intimidation to get your current or previous partner to have sex with you when they did not want to?	1	2	3	4
C	In the past 6 months, how many times have you forced a current or previous partner to do something else sexual that they did not want to do?	1	2	3	4

**Economic IPV - Perpetration**					
A	In the past 6 months how often did you stop your partner from getting a job, going to work, trading or earning money?	NEVER	ONCE	FEW	MANY
B	In the past 6 months how often did you take your partner's earnings against their will?	1	2	3	4
C	In the past 6 months how often did you throw your partner out of the house?	1	2	3	4
D	In the past 6 months how often did you lock your partner in a house or room so they could not leave?	1	2	3	4
E	In the past 6 months how often did you spend money on alcohol, tobacco or other things for yourself when you knew you did not have enough for essential household expenses?	1	2	3	4

SECTION F: GENDER ATTITUDES

Attitudes about relations between men and women

For each of the following statements please say answer whether you strongly agree, agree, disagree or strongly disagree with the following statements:					
		Strongly Disagree	Disagree	Agree	Strongly Agree
A	I think that a woman should obey her husband	1	2	3	4
B	I think that a man should have the final say in all family matters	1	2	3	4
C	I think that a woman needs her husband's permission to do paid work	1	2	3	4
D	I think that a woman cannot refuse to have sex with her husband.	1	2	3	4
E	I think if a woman does not physically fight back, it's not rape	1	2	3	4
F	I think that there is nothing a woman can do if her husband wants to have girlfriends	1	2	3	4
G	I think that men should share the work around the house with women such as doing dishes, cleaning and cooking	1	2	3	4
H	I think that children belong to a man and his family	1	2	3	4
I	I think that if a wife does something wrong her husband has the right to punish her	1	2	3	4
J	I think that if a man has paid Lobola for his wife, he owns her.	1	2	3	4
K	I think that if a man has paid Lobola for his wife, she must have sex when he wants it	1	2	3	4
L	I think that if a man beats you it shows that he loves you.	1	2	3	4

SECTION G: GENDER EQUALITY

Sexual relationship power scale

The next set of statements are about your relationship with **<u>your current or most recent main</u>** partner, please say for each if you strongly agree, agree, disagree or strongly disagree:					
	RELATIONSHIP CONTROL SCALE	Never	Rarely	Sometimes	Almost Always
A	When my partner wants sex, they expect me to agree	1	2	3	4
B	If I asked my partner to use a condom, they would get angry.	1	2	3	4
C	My partner won't let me wear certain things.	1	2	3	4
D	When my partner and I disagree or get in an argument, they get their way.	1	2	3	4
E	My partner tells me who I can spend time with.	1	2	3	4
F	When I wear certain clothes or dress up my partner thinks I may be trying to attract other partners	1	2	3	4
G	My partner constantly asks where I am or monitors my behaviours and whereabouts e.g. makes several calls in an hour to find out where I am or follows me when I leave the house.	1	2	3	4
H	My partner threatens to leave me if they don't get their way	1	2	3	4
I	My partner checks my phone to monitor who I am talking to.	1	2	3	4
J	My partner sets rules about where I can go and when I should be home.	1	2	3	4
K	My partner constantly comments and criticises me and my lifestyle	1	2	3	4
L	My partner sets rules about who I can have fun with	1	2	3	4
M	My partner likes to choose who I can be friends with	1	2	3	4

Household Decision-Making Control for Women and Girls

	How much control do you have over your decision?	How much control do your partner or other household members have over your decision?	If your partner or family do not agree with your decision, can any of the following happen?
1. Leaving the house to go into the community	A. None<br>B. Very Little<br>C. Some<br>D. A fair amount<br>E. Full Control	A. None<br>B. Very Little<br>C. Some<br>D. A fair amount<br>E. Full Control	A. They will stop you.<br>B. They will be angry and may speak badly about you.<br>C. They will punish you.<br>D. Nothing will happen.
2. Who you will associate with outside of your household	A. None<br>B. Very Little<br>C. Some<br>D. A fair amount<br>E. Full Control	A. None<br>B. Very Little<br>C. Some<br>D. A fair amount<br>E. Full Control	A. They will stop you.<br>B. They will be angry and may speak badly about you.<br>C. They will punish you.<br>D. Nothing will happen.
3. From whom to seek health care for yourself	A. None<br>B. Very Little<br>C. Some<br>D. A fair amount<br>E. Full Control	A. None<br>B. Very Little<br>C. Some<br>D. A fair amount<br>E. Full Control	A. They will stop you.<br>B. They will be angry and may speak badly about you.<br>C. They will punish you.<br>D. Nothing will happen.
4. When to seek health care for yourself	A. None<br>B. Very Little<br>C. Some<br>D. A fair amount<br>E. Full Control	A. None<br>B. Very Little<br>C. Some<br>D. A fair amount<br>E. Full Control	A. They will stop you.<br>B. They will be angry and may speak

			badly about you.<br>C. They will punish you.<br>D. Nothing will happen.
5. Large household purchases	A. None<br>B. Very Little<br>C. Some<br>D. A fair amount<br>E. Full Control	A. None<br>B. Very Little<br>C. Some<br>D. A fair amount<br>E. Full Control	A. They will stop you.<br>B. They will be angry and may speak badly about you.<br>C. They will punish you.<br>D. Nothing will happen.
6. Visits to family or relatives	A. None<br>B. Very Little<br>C. Some<br>D. A fair amount<br>E. Full Control	A. None<br>B. Very Little<br>C. Some<br>D. A fair amount<br>E. Full Control	A. They will stop you.<br>B. They will be angry and may speak badly about you.<br>C. They will punish you.<br>D. Nothing will happen.

SECTION H: EARNINGS IN THE PAST MONTH

Labour and household economic status

Question	Response
1. What do you do to make money?	☐ Employed<br>☐ Self-employed<br>☐ Temp/casual worker<br>☐ Do odd jobs<br>☐ Unemployed
2. If you get money from a job, can you please indicate approximately how much you earn in a month?	R________________<br><br>☐ Refused
3. Can you indicate any other money (e.g., grants, family members,	________________________________

organisations, partners, learnerships, maintenance, borrowing money or other activities)	☐ Refused
4. If you are not working, what is the main reason for this? (Tick all that apply)	☐ COVID-19 Lockdown related<br>☐ Poor health<br>☐ Retrenched<br>☐ Retired/too old/pensioner<br>☐ Pregnant/maternity<br>☐ Disability<br>☐ Have never worked<br>☐ Looting<br>☐ Other (specify below)<br>________________<br>☐ Refused
5. If you worked before, but are now no longer working, when was the last time that you worked?	☐ Less than 3 months ago<br>☐ 3 to 6 months ago<br>☐ 6 to 9 months ago<br>☐ 9 to 12 months ago<br>☐ more than a year ago
6. How much of money (ZAR) is spent every month by your household on:	
i. Food	R________________
ii. Transport *(e.g. to work, school, to look for jobs, doing to hospital/clinic GP, etc.)*	R________________
iii. Healthcare-related products or services *(e.g. visits to the doctor, hospital / clinic visits, medication, etc.)*	R________________
iv. Water	R________________
v. Electricity	R________________
vi. Other important household things	R________________
7. Can you please tell me what some of those other things are?	i. ________________<br>ii. ________________<br>iii. ________________<br>iv. ________________<br>v. ________________
8. Total household expenditure (ZAR)	R________________

SECTION I: HOUSEHOLD AND SOCIAL OUTCOMES

Question	Response
1. How many government grants does your household (including yourself) receive?	Number: _______________<br><br>☐ Refused<br>☐ Don't know
2. Please tick all the government grants received by your household (including yourself)	☐ Child Support Grant<br>☐ Old Age Pension Grant<br>☐ Disability Grant<br>☐ Foster Child Grant<br>☐ Care Dependency Grant<br>☐ Other (*Specify below)*<br>_______________<br><br>☐ Refused
3. Do you or your household get any other form of income?	☐ Yes<br>☐ No
4. Please indicate all the sources of income that your household receives (*Please tick all that apply*)	☐ Income from employment<br>☐ Income from business<br>☐ Government grants<br>☐ Money from friends and family<br>☐ Pension<br>☐ Other (*Specify below*)<br>_______________<br><br>☐ Refused
5. What is the combined income of your household (i.e. if you add together what people in the house each contribute)?	R: _______________<br><br>☐ Refused<br>☐ Don't know
6. In the past month, did you or your household receive food or shelter from any of these sources?	☐ Government<br>☐ NGO's, churches, and other associations<br>☐ Neighbours or community<br>☐ Other (*Please specify*)<br>_______________
7. Do you frequently take any member of your family to a healthcare facility or collect medication for them?	☐ Yes<br>☐ No
8. If yes, how much of your time was spent doing this in the past month?	☐ less than 4 hours<br>☐ 4 to 8 hours<br>☐ 8 to 12 hours<br>☐ More than 12 hours

Questions relating specifically to costs associated with taking care of CALHIV

**i. Direct Costs:**	
**Question**	**Response**
**In the past month, how much have you spent on…**	
1. Transport taking your child to and from health facilities (e.g. clinic, hospital, general practitioner, etc.)?	R: ________________
2. Admission and consultation fees?	R: ________________
3. Medication?	R: ________________
4. Food while at the health facility?	R: ________________
5. Any fees paid for diagnostic procedures?	R: ________________
6. Cost for somebody else accompanying your child to healthcare facility other than yourself?	R: ________________
7. Cost for somebody else taking care of your child when you are not around (e.g. at work)?	R: ________________
8. Total direct costs	R: ________________
**ii. Indirect Costs:**	
**In the past month…**	
9. How often were you unable to carry out your normal daily activities AT ALL due to your child being ill (e.g. (in)formal work, doing chores, etc.)?	Days: ________________
10. How often were you unable to care out your normal daily activities for a few hours due to your child being ill?	Days: ________________
Coping Strategies:	
**In the past month…**	
11. Can you please indicate how you have been able to cope with looking after the healthcare of your child	☐ Borrowing interest free money<br>☐ Borrowing interest baring money<br>☐ Loanshark<br>☐ Help from neighbours or community<br>☐ Family labour substitution<br>☐ Selling assets<br>☐ Children stop going to school<br>☐ Family contributions<br>☐ Social grants<br>☐ Other (*please specify*)

	________________

SECTION J: FOOD INSECURITY EXPERIENCE SCALE (FIES): Household Referenced

Statement	No	Yes	Don't know	Refused to answer
1. You or others in your household worried about not having enough food to eat because of a lack of money or other resources?				
2. Was there a time when you or others in your household were unable to eat healthy and nutritious food because of a lack of money or other resources?				
3. Was there a time when you or others in your household ate only a few kinds of foods because of a lack of money or other resources?				
4. Was there a time when you or others in your household had to skip a meal because there was not enough money or other resources to get food?				
5. Was there a time when you or others in your household ate less than you thought you should because of a lack of money or other resources?				
6. Was there a time when your household ran out of food because of a lack of money or other resources?				
7. Was there a time when you or others in your household were hungry but did not eat because there was not enough money or other resources for food?				
8. Was there a time when you or others in your household went without eating for a whole day because of a lack of money or other resources?				

SECTION K: EVERYDAY DISCRIMINATION SCALE (Contracted)

Statement	Almost everyday	At least once a week	A few times a month	A few times a year	Less than once a year	Never
You are treated with less courtesy or respect than other people are.						
You receive poorer service than other people at restaurants or stores.						
People act as if they think you are not smart.						
People act as if they are afraid of you.						
You are threatened or harassed.						

SECTION L: MULTIDIMENSIONAL SUPPORT SCALE OF PERCEIVED SOCIAL SUPPORT (MSPSS)

	Very Strongly Disagree	Strongly Disagree	Mildly Disagree	Neutral	Mildly Agree	Strongly Agree	Very Strongly Agree
1. There is a special person who is around when I am in need.	1	2	3	4	5	6	7
2. There is a special person with whom I can share my joys and sorrows.	1	2	3	4	5	6	7
3. My family tries to help me.	1	2	3	4	5	6	7

4. I get the emotional help & support I need from my family.	1	2	3	4	5	6	7
5. I have a special person who is a real source of comfort to me.	1	2	3	4	5	6	7
6. My friends really try to help me.	1	2	3	4	5	6	7
7. I can count on my friends when things go wrong.	1	2	3	4	5	6	7
8. I can talk about my problems with my family.	1	2	3	4	5	6	7
9. I have friends whom I can share my joys and sorrows.	1	2	3	4	5	6	7
10. There is a special person in my life who care about my feelings.	1	2	3	4	5	6	7
11. My family is willing to help me make decisions.	1	2	3	4	5	6	7
12. I can talk about my problems with my friends.	1	2	3	4	5	6	7

SECTION M: CLOSURE & FIELDWORKERS COMMENTS

Narration:<br>• Thank you for completing this activity with me.<br>• I appreciate your time.<br>• The information you have given us will not be shared with others, will not contain your name and will be stored in a locked cupboard.<br>• If you have any questions about the research we are doing or your participation in this study, or if you would like more information about any of the topics we have discussed in this interview, please feel free to contact us on the cellphone number listed on the study card you were given.<br>• Is there anything else you would like to share with us?<br>• [Refer as needed]
Note to the fieldworker: **Please kindly complete this section**
Please write down anything unusual about this interview, your impressions of the participant or the interview, concerns you might have (referral needed), or anything else that might help us to understand the information you have collected?

1. What are some of the key challenges you experienced with administering the questionnaire	
a. Kindly detail any question/module that was confusing for either you/participant	
b. Were there any response options that were unclear?	
2. What were some of the specific questions/comments from the participant?	
3. Describe your experience with administering this questionnaire:	
4. On average, how long did it take to complete the questionnaire?	
5. How would you improve the questionnaire?	
