# Supplementary material for: A cash transfer plus gender transformative economic empowerment intervention seeking to improve the wellbeing of caregivers of children and adolescents living with HIV in South Africa: a feasibility study protocol for a pilot cluster randomized trial
Source: Pilot Feasibility Stud. 2025 Apr 23;11:52. doi: 10.1186/s40814-025-01643-3 (PMC12020082; doi:10.1186/s40814-025-01643-3)
Supplement: Supplementary file 4 — Additional file 4: Appendix D – Proposed Theories of Change. Proposed Theories of Change for CWeL and SSCF trials. [file 40814_2025_1643_MOESM4_ESM.pdf]

Appendix D

Proposed Theories of Change

Table D1. CWEL Logic model and Theory of Change

Context	Inputs	Outputs	Outcomes
Limited staff training and experience with telephonic consent and data collection, randomisation, and electronic administration of economic incentives	**Trial activities:**<br>- Training of field staff<br>- Implementation of an electronic data collection and intervention delivery system<br>- Monitoring of trial activities by co-co-principal investigators and field supervisor	**Trial-related**:<br>- All staff adequately trained on trial SOPs<br>- Increase in staff confidence and skills with screening, consent randomisation, electronic data entry, follow-up and intervention delivery systems (ABSA Cash-Send, SMS)	**Primary outcomes:**<br>- Consent rate (≥ 80%)<br>- Retention rate (≥ 75%)<br>- Protocol adherence (≥ 90%)
**Potential determinants of caregiver QoL:**<br>- Age, education and employment status, HIV status, depressive symptom score, household socio- economic status<br>- Caregiving responsibilities and financial constraints, stigma, social isolation, family functioning, coping	**Economic incentive**<br>- Cash incentive (R1050)<br>- SMS reminders promoting wellbeing	- Reduced self-perceived financial burden<br>- Improved sense of purpose and meaning<br>- Improved relational ties with ALHIV<br>- Improved caregiver depressive symptom score	**Secondary outcome**<br>- Increase in overall caregiver wellbeing

Table D2. SSCF theory of change (27)

Interventions	Creating futures		Stepping stones					
Overall result	REDUCED VAWG AND OTHER OUTCOMES							
Hypothesised effect	Greater engagement in income generating activity gives women more social power and enhances men's selfworth		Critical reflection methodology challenges acceptance of patriarchy, opens doors to more respectful masculinities and more assertive femininities, all leading to less VAWG			Empowerment overall and better relationships enhance mood and reduces substance abuse		Empowering methodology and group work combined with communication skills improves relationships and handling of disagreements
Required intervention elements	Life skills intervention to assist income generation or return to education		Focus on building and understanding of gender equity and tackling VAWG					Building communication skills combined with gender equity
Amenable risk and aggrevating factors	Low economic power	Low education	Masculinities predicated on dominance over & control of women	Women's acceptance of patriarchal dominance	Ingrained acceptability of use of VAWG	Depression	Men's substance abuse	Poor relationship skills (esp.over conflict)
MEN AND WOMEN								
Problem statement	One third of South African women experience VAWG in their lifetime and this is higher in some local areas. The problem is driven substantially by the low status and power of girls and women, and social norms related to masculinity which emphasise dominance and control over women. In informal settlements the situation is exacerbated by very high youth unemployment and generally low levels of education (nationally 60% of learners fail to reach matric).							
